# Double Positive CD4^+^CD8^+^ T Cells Are Enriched in Urological Cancers and Favor T Helper-2 Polarization

**DOI:** 10.3389/fimmu.2019.00622

**Published:** 2019-03-29

**Authors:** Perrine Bohner, Mathieu F. Chevalier, Valérie Cesson, Sonia-Christina Rodrigues-Dias, Florence Dartiguenave, Rodolfo Burruni, Thomas Tawadros, Massimo Valerio, Ilaria Lucca, Denise Nardelli-Haefliger, Patrice Jichlinski, Laurent Derré

**Affiliations:** Urology Research Unit, Urology Department, University Hospital of Lausanne (CHUV), Lausanne, Switzerland

**Keywords:** CD4^+^CD8^+^ T cells, double positive T cells, Th2, Th1, bladder cancer, prostate cancer, kidney cancer

## Abstract

The immune system plays a central role in cancer development, showing both anti-tumor and pro-tumor activities depending on the immune cell subsets and the disease context. While CD8 T cells are associated with a favorable outcome in most cancers, only T helper type 1 (Th1) CD4 T cells play a protective role, in contrast to Th2 CD4 T cells. Double positive (DP) CD4^+^CD8^+^ T cells remain understudied, although they were already described in human cancers, with conflicting data regarding their role. Here, we quantified and phenotypically/functionally characterized DP T cells in blood from urological cancer patients. We analyzed blood leukocytes of 24 healthy donors (HD) and 114 patients with urological cancers, including bladder (*n* = 54), prostate (*n* = 31), and kidney (*n* = 29) cancer patients using 10-color flow cytometry. As compared to HD, levels of circulating DP T cells were elevated in all urological cancer patients, which could be attributed to increased frequencies of both CD4^high^CD8^low^ and CD4^+^CD8^high^ DP T-cell subsets. Of note, most CD4^high^CD8^low^ DP T cells show a CD8αα phenotype, whereas CD4^+^CD8^high^ cells express both CD8α and CD8β subunits. Functional properties were investigated using *ex-vivo* generated DP T-cell clones. DP T cells from patients were skewed toward an effector memory phenotype, along with enhanced Th2 cytokine production. Interestingly, both CD8αα and CD8αβ DP T cells were able to trigger Th2 polarization of naïve CD4 T cells, while restraining Th1 induction. Thus, these data highlight a previously unrecognized immunoregulatory mechanism involving DP CD4^+^CD8^+^ T cells in urological cancers.

## Introduction

The crucial role of anti-tumor immune responses in cancer patients has been intensively studied during the last two decades, leading to unprecedented opportunities to effectively treat several malignancies (1–4). However, mechanisms hindering efficient immune responses to human tumors still need to come under closer scrutiny, and the complex interplay between various immune cell-subsets requires being well-deciphered to improve or design novel immunotherapeutic strategies. CD8^+^ T cells and type-1 helper CD4^+^ T cells (Th1) are thought to play a major role in effective anti-tumor responses, and thus associate to good prognosis in most human cancers (4, 5). In contrast, Th2 CD4^+^ T cells—which are involved in humoral immunity by providing B-cell help—are more often associated to poor prognosis (5). This detrimental effect is thought to be mostly attributed to cross-regulation between Th subsets (6), allowing Th2 cells to polarize immune cells away from a protective anti-tumor type-1-immunity (7, 8).

The role of CD4^+^CD8^+^ double positive (DP) T cells is largely understudied. Indeed, DP T cells were primarily considered as a developmental stage in the thymus, before maturation as either CD4^+^ or CD8^+^ (single positive) mature T cells (9). Thus, in peripheral blood and tissues, most T cells have retained expression of only one of these co-receptors corresponding to different functions, with CD8 T cells mostly involved in cytotoxicity toward infected or tumor cells, and CD4 T cells playing helper functions to orchestrate the immune response. However, mature CD4^+^CD8^+^ double positive T cells have been described in the peripheral blood and tissues in various settings, including in human cancers (10). The conflicting literature regarding the role of DP T cells—cytotoxic vs. immunosuppressive (10)—may indicate that these cells are heterogeneous and/or show pleiotropic functions that need to be investigated in each particular disease context. DP T cells have never been studied in patients with urological cancer. Here, we quantitatively and phenotypically described DP T cells in blood from patients with bladder, prostate and kidney cancers. We also investigated their immunomodulatory functions and found that DP T cells favor the polarization of naïve CD4^+^ T cells into a Th2 functional profile. This previously unrecognized capacity of DP T cells was observed in healthy donors (HD) and exacerbated in patients with urologic cancer, who moreover showed elevated levels of circulating DP T cells.

## Materials and Methods

### Study Population

Fifty four patients with bladder cancer, 29 patients with kidney cancer, and 31 patients with prostate cancer (mostly male; Table 1) were recruited at the Lausanne University Hospital. Peripheral blood was collected in heparin-containing tubes. Fresh peripheral blood mononuclear cells (PBMCs) were purified by density gradient centrifugation (Isopaque-Ficoll) within 2 h after blood sampling. Cells were cryopreserved in RPMI medium 1640 supplemented with 40% FCS and 10% DMSO. Blood samples were also collected from 24 healthy volunteers (>50 years old and 80% of male) through the local Swiss blood bank.

**Table 1:** Characteristics of urological cancer patients.

**Characteristics**	**Bladder**	**Prostate**	**Kidney**
N° of patients	54	31	29
Age, yr, median (IQR)	70 (62.3–80)	66 (60.5–70.5)	65 (58–69)
**SEX**, ***n*** **(%)**			
Male	42 (77.8)	31 (100)	23 (79.3)
Female	12 (22.2)	n.a.	6 (20.7)
**TUMOR STATUS**, ***n*** **(%)**			
pTa	18 (33.3)	n.a.	n.a.
pT1	12 (22.2)	3 (9.7)	12 (41.4)
pT2	5 (9.3)	17 (54.8)	2 (6.9)
pT3	13 (24.1)	10 (32.3)	14 (48.3)
pT4	1 (1.8)	1 (3.2)	1 (3.4)
CIS alone	5 (9.3)	n.a.	n.a.
**DRAINING LYMPH NODE STATUS**, ***n*** **(%)**			
Nx	1 (1.8)	2 (6.5)	7 (24.1)
N0	48 (88.9)	28 (90.3)	20 (69)
N1	5 (9.3)	1 (3.2)	0 (0)
>N2	0	0	2 (6.9)
**GLEASON**			
6	n.a.	6 (19.3)	n.a.
7	n.a.	22 (71)	n.a.
8	n.a.	0 (0)	n.a.
9	n.a.	2 (6.5)	n.a.
10	n.a.	1 (3.2)	n.a.
**FURHMAN**			
I	n.a.	n.a.	0 (0)
II	n.a.	n.a.	11 (37.9)
III	n.a.	n.a.	10 (34.5)
IV	n.a.	n.a.	6 (20.7)
not determined	n.a.	n.a.	2 (6.9)

*n.a, not applicable*.

Bladder and kidney tumor tissue samples were carefully collected by pathologist from cystectomy or nephrectomy, respectively. Tissue samples were then put in a Petri dish containing RPMI 1640 with 1 μg/mL DNAse I (Sigma Aldrich), cut into small pieces using sterile scissors, transferred into a sterile strainer and grinded with a syringe plunger in another Petri dish. The dissociated tissue was rinsed and transferred in a conical tube through a 40 μm filter (Fisher Scientific). After centrifugation, cells were resuspended in fresh medium, counted and were subsequently used for flow cytometry analysis.

All patients provided written informed consent prior to their participation in the study. This study was approved by the ethics committee of the canton of Vaud, Switzerland (protocol #119/10).

### Flow-Cytometry Analyses

The following mAbs were used at predetermined optimal concentrations: anti–CCR7-PE, anti–CD3-PeCy7, anti–CD14-FITC, anti–CD8α-PEAF610 (Invitrogen); anti–CD4-APC/H7 (BD Pharmingen); anti–CD8β-eF660, anti–CD45RA-eF450, anti–IL4-PeCy7 (eBioscience). FcR Blocking Reagent (Miltenyi Biotec) was used to block unwanted binding of antibodies and increase the staining specificity of cell surface antigens. Cells were stained for surface antigens at 4°C for 20 min, and an amine reactive dye (Aqua LIVE/DEAD Stain Kit; Thermo Fisher Scientific) was used for dead cell exclusion according to the manufacturer's instructions. A representative example of the full gating strategy is shown on Supplementary Figure 1A. When intracellular staining was required, cells were then fixed and permeabilized using the “Intracellular Fixation and Permeabilization Buffer Set” (eBioscience) according to the manufacturer's recommendations. Cytokine staining was then performed using anti–INF-ɤ-BV421 (BioLegend) and anti–IL-4-PeCy7 (eBioscience) for 30 min at room temperature. Sample acquisition was performed on the Gallios Flow Cytometer (Beckman Coulter). Analyses were performed using the FlowJo software (TreeStar).

### Generation of DP T-Cell Clones

DP T cells were sorted by FACS sorting (FACSARIA III, BD Biosciences). Sorted cells were cloned by limiting dilution, and expanded in RPMI medium 1640 supplemented with 8% human serum, 150 U/mL recombinant human IL-2 (rIL-2, from Proleukin), 1 μg/mL phytohemagglutinin (PHA), and irradiated allogeneic PBMC (3,000 rad) as feeder cells (feeders to DP T cells ratio of 2:1). Successfully expanded clones were checked for purity (only those with >90% DP T cells were selected) and cryopreserved for subsequent functional assays. T-cell suppression assay was performed as we previously described (11, 12).

### Bead-Based Multiplex Assay for the Measurement of Cytokine Secretion by DP T Cells

DP T-cell clones (30,000 cells/well in a 96 well-plate) were stimulated during 24 h with plate-bound anti-CD3 antibody (pre-coated at 2 μg/mL overnight at 4°C) and soluble anti-CD28 (2 μg/mL). Cell-free supernatants were collected and cytokine concentrations (IFN-γ, TNF-α, IL-4, and IL-5) were measured using a Luminex assay according to the manufacturer's instructions (Thermo Fisher Scientific). Supernatants were also used for naïve CD4 T-cell conditioning assay as described below.

### Th1 vs. Th2 Induction From Naive T Cells Following Conditioning With DP T-Cell Supernatants

Naïve CD4^+^ T cells (CD3^+^CD4^+^CD8^neg^CD45RA^+^CCR7^+^) from healthy donors were FACS-sorted (FACSARIA III, BD Biosciences) and stimulated for 48 h with plate-bound anti-CD3 and soluble anti-CD28, in the presence of above-described supernatants (dilution 1:1 with fresh medium) of stimulated DP T-cell clones from patients or healthy donors. Supernatants from non-stimulated corresponding DP T-cell clones were used as a control. Cells were then transferred into a new plate and fresh supernatant was added. Following a 4 day-incubation, cells were stimulated for 6 h with PMA/Ionomycin (Cell Stimulation Cocktail, eBioscience) in the presence of monensin and Brefeldin A (Protein Transport Inhibitors Cocktail, eBioscience). IFN-γ and IL-4 expression was measured by intracellular cytokine staining (as described above). Experiment scheme is depicted in **Figure 4A**.

### Statistics

Statistical comparisons were evaluated using *t*-tests or non-parametric Mann-Whitney (and Wilcoxon, for paired values) tests, when data passed or failed normality test, respectively. For multiple comparisons, Kruskal-Wallis test was performed, followed by Dunn's *post-hoc* correction for multiple comparison. A *p* < 0.05 was considered statistically significant. All statistical analyses were performed using GraphPad Prism, version 7 (GraphPad Software).

## Results

### Identification of DP T Cells in Patients With Urological Cancers

**Figure 1:**
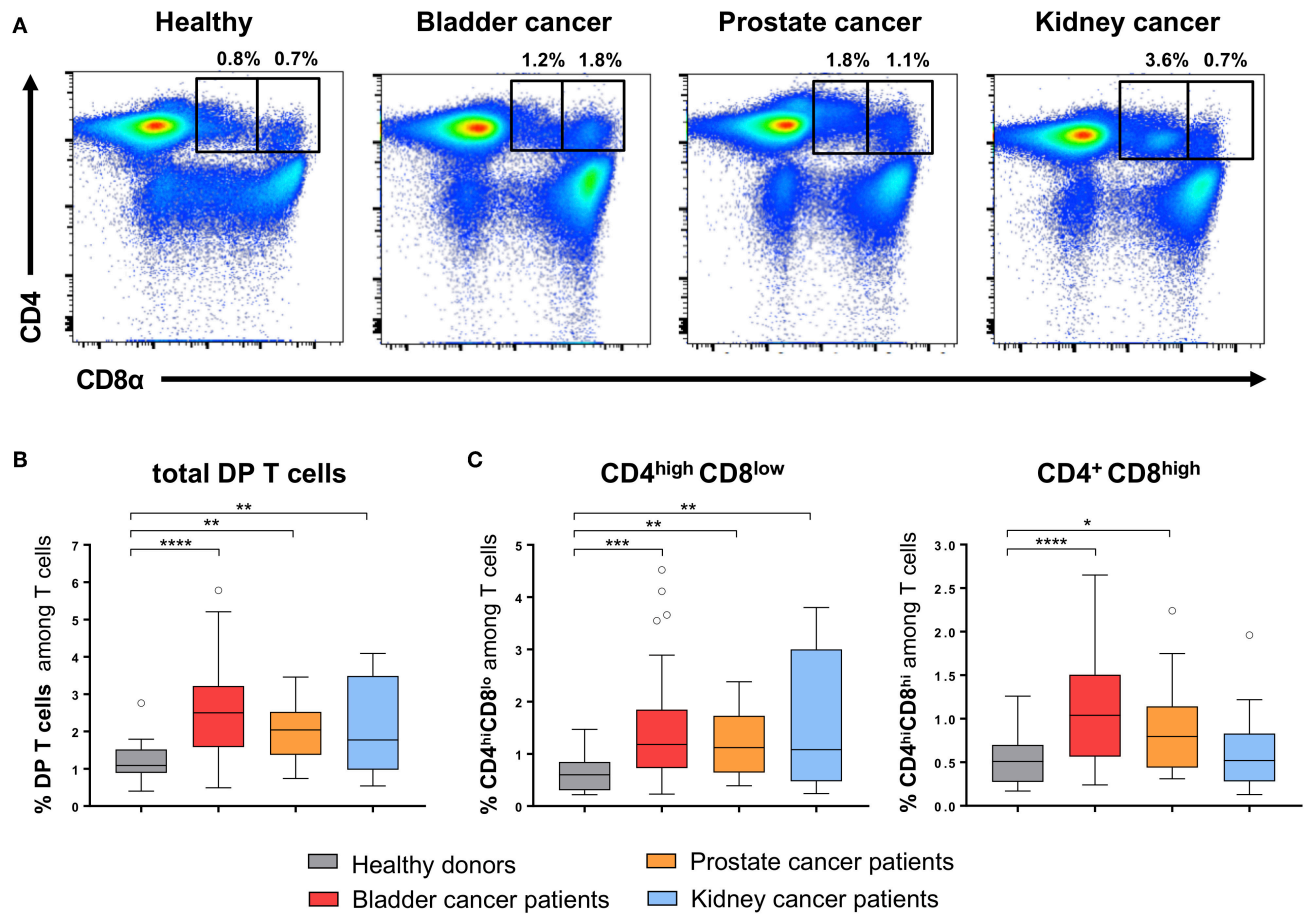
Quantification of CD4^+^CD8^+^ DP T cells in urological cancer patients. **(A)** Representative example of CD4^high^CD8^low^ and CD4^+^CD8^high^ DP T cells gated on live CD3^+^ T cells in PBMC from HD and urological patients. Frequencies of total DP T cells **(B)** and of the two DP T-cell subpopulations **(C)** in PBMCs from HD (*n* = 24) and urological cancer patients: bladder (*n* = 54), prostate (*n* = 31) and kidney cancer (*n* = 29). *p ≤ 0.05; **p ≤ 0.01; ***p ≤ 0.001; ****p ≤ 0.0001.

Using flow-cytometry analysis, we identified CD4^+^CD8^+^ double positive (DP) T cells in peripheral blood mononuclear cells (PBMCs) from HD and from patients (Table 1) with bladder, prostate or kidney cancers (Figures 1A,B). According to the CD8 expression level, we defined two subpopulations of DP T cells: CD4^high^CD8^low^ and CD4^+^CD8^high^ (Figures 1A,C). Of note, the resolution of the labeling did not allow to reliably distinguish CD4^high^CD8^high^ from CD4^low^CD8^high^ DP T cells (Figure 1A) within the CD4^+^CD8^high^ population (13). Nevertheless, the frequency of total DP T cells (Mean percentage ± SEM of 1.18 ± 0.12 for HD; 2.68 ± 0.19 for bladder; 1.99 ± 0.13 for prostate; 3.26 ± 0.77 for kidney) was significantly elevated in all urologic cancers as compared to healthy controls (Figure 1B), independent of tumor stage or grade (*data not shown*). This increase was ascribed to elevated levels of both CD4^high^CD8^low^ and CD4^+^CD8^high^ subsets, except in kidney cancer where the increase in DP T cells was only attributed to the CD4^high^CD8^low^ subset (Figure 1C). These alterations in DP T-cell frequencies were not owing to a variation in CD4^+^ or CD8^+^ single positive T-cells as their frequency were similar between HD and cancer patients (Supplementary Figure 1B). Also, the frequency of total CD3^+^ T cells was not altered in the blood from cancer patients (Supplementary Figure 1C).

### Memory/Differentiation Phenotype of DP T Cells

**Figure 2:**
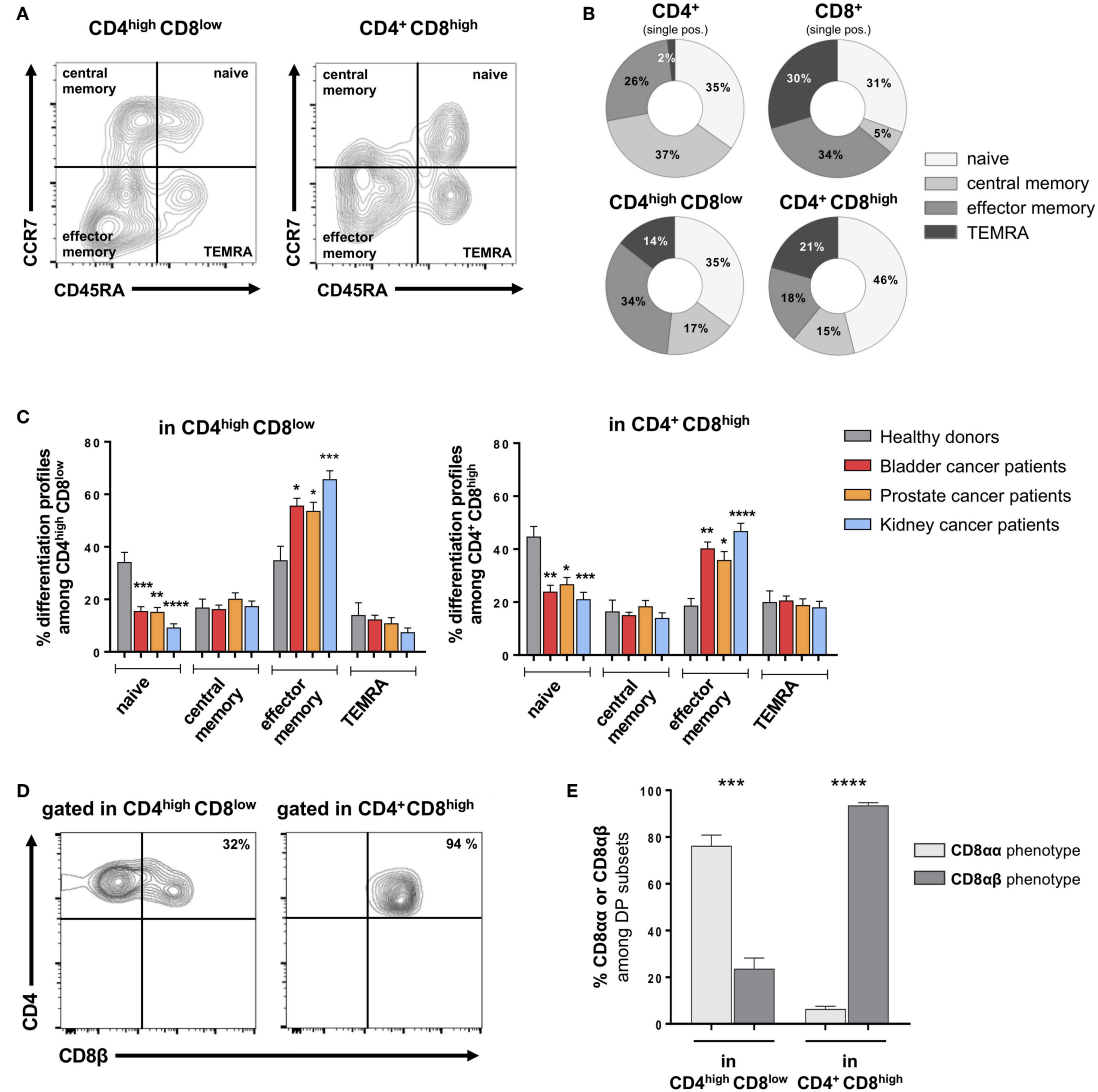
Alterations in memory subset distribution among DP T cells from urological cancer patients. **(A)** Representative example of differentiation phenotype, as defined by CD45RA and CCR7 labeling of CD4^high^CD8^low^ and CD4^+^CD8^high^ DP T cells gated on live CD3^+^ T cells; naïve: CD45RA^+^CCR7^+^; central memory: CD45RA–CCR7^+^; effector memory: CD45RA^+^CCR7^+^; and terminally differentiated effector memory (TEMRA): CD45RA^+^CCR7^−^. **(B)** Differentiation stage distribution in DP and conventional single-positive T cells from healthy donors (HD). **(C)** Comparison between urological cancer patients and HD for each the memory subsets frequency among DP T cells (mean ± SEM). **(D)** Representative example of CD8β labeling in CD4^high^CD8^low^ and CD4^+^CD8^high^ from PBMC. **(E)** Proportion (mean ± SEM) of cells with a CD8αα and CD8αβ phenotype in CD4^high^CD8^low^ and CD4^+^CD8^high^ DP T-cell subsets (*n* = 10 HD). *p ≤ 0.05; **p ≤ 0.01; ***p ≤ 0.001; ****p ≤ 0.0001.

The differentiation profile of DP T cells was assessed by the analysis of CCR7 and CD45RA expression (14, 15), allowing the identification of naïve, central memory, effector memory and terminally differentiated effector memory cells re-expressing CD45RA (TEMRA) (Figure 2A). In HD, CD4^+^CD8^high^ and CD4^high^CD8^low^ DP T cells showed quite similar differentiation profiles, which seem intermediate between CD4 and CD8 single-positive T cells (Figure 2B). Strikingly, both DP T-cell subsets from cancer patients showed a differentiation profile skewed toward the effector memory phenotype, along with a shortening of the naïve compartment, as compared to HD (Figure 2C). Notably, this profile was consistently and significantly observed in bladder, prostate as wells as kidney cancers.

**Figure 3:**
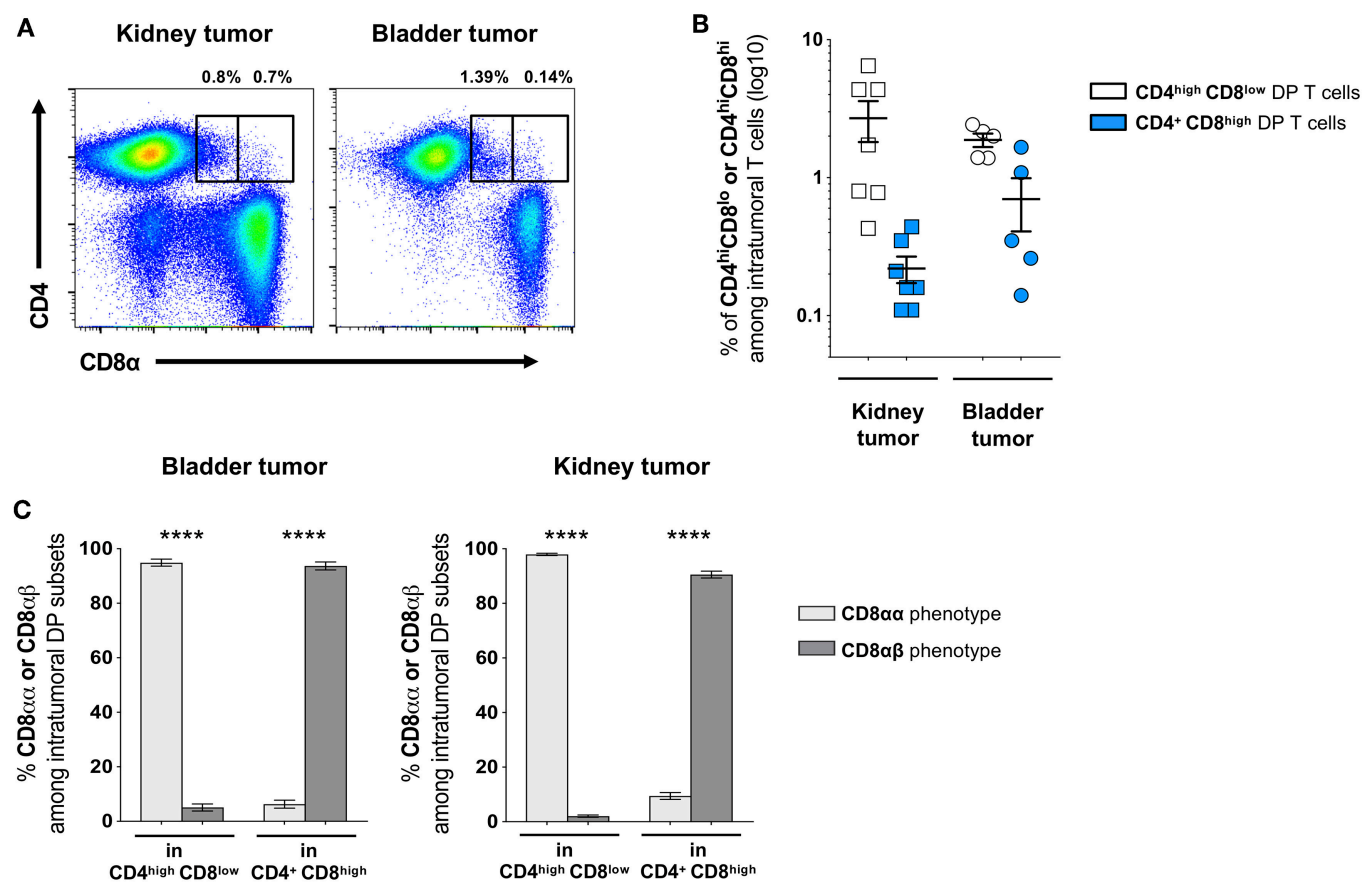
Frequency of DP T cells in bladder and kidney tumors. **(A)** Representative example of CD4^high^CD8^low^ and CD4^+^CD8^high^ DP T cells gated on live CD3^+^ T cells in freshly dissociated kidney and bladder tumors. **(B)** Frequencies of intratumoral CD4^high^CD8^low^ and CD4^+^CD8^high^ DP T cells and **(C)** their CD8α/CD8β phenotype (mean ± SEM). ****p ≤ 0.0001.

To further describe the DP T-cell phenotype, we next analyzed the phenotype of the CD8 subunits. Indeed, studies revealed that DP T cells with immunosuppressive function show a peculiar CD8αα – instead of the conventional CD8αβ – phenotype (16, 17). In healthy donors, the CD4^+^CD8^high^ subset showed a conventional CD8αβ phenotype, while a majority of CD4^high^CD8^low^ cells did not express the CD8β subunit thus showing the peculiar CD8αα phenotype (Figures 2D,E). Similar profiles were found in patients as in healthy donors (*data not shown*). Furthermore, we analyzed the presence of DP T cells in fresh bladder and kidney tumor tissue from surgical specimens recovered after cystectomy or nephrectomy, respectively (Figure 3A). Intratumoral CD4^high^CD8^low^ and CD4^+^CD8^high^ DP T cells were detected at variable frequencies (Figure 3B) and, as found in circulating DP T cells, showed a CD8αα or CD8αβ phenotype, respectively (Figure 3C).

### Th1 and Th2 Cytokine Production by DP T Cells

**Figure 4:**
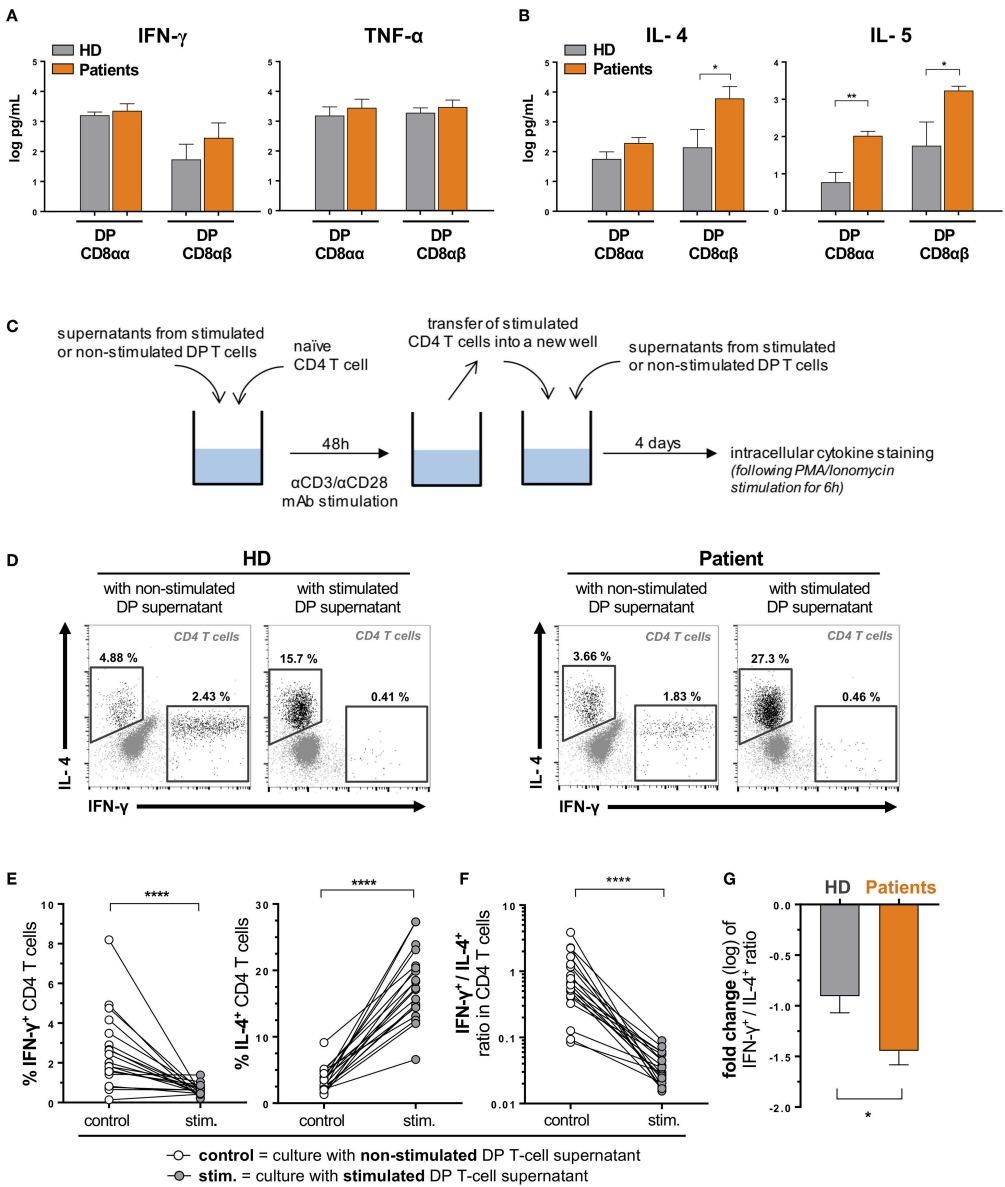
DP T cells favor a Th2 over Th1 polarization of naïve CD4 T cells. DP T-cell clones were generated from *ex vivo* sorted PBMCs of healthy donors (HD) and urological cancer patients. Clones (3 CD8αα and 4 CD8αβ clones from 3 HD and 6 CD8αα and 4 CD8αβ clones from one patient of each cancer, i.e., bladder, prostate and kidney) were *in vitro* stimulated (anti-CD3/anti-CD28) for 48 hours, and concentrations (mean ± SEM) of **(A)** Th1 (IFN-γ, TNF-α) and **(B)** Th2 (IL-4, IL-5) cytokines were measured in supernatants by Luminex. **(C)** Experimental procedure. Representative examples **(D)** and percentages **(E)** of IL-4 and IFN-γ expression by polarized CD4^+^ naïve T cells (from 2 HD) upon stimulation in the presence of supernatants from DP T cell clones from HD or patients (described in **A,B**). **(F)** Ratio between IFN-γ- and IL-4-expressing CD4^+^ T cells upon stimulation is shown and **(G)** variation (fold change) of this ratio (mean ± SEM) compared when using DP T cells from patients and HD. *p ≤ 0.05; **p ≤ 0.01; ****p ≤ 0.0001.

As DP T cells have a high capacity to produce cytokines (18) and are moreover enriched in effector memory cells in patients with urological cancers, we next interrogated the cytokine secretion profile of DP T cells from patients compared to HD. To this purpose, we sorted DP T cells according to CD8α and β expression (in order to increase the accuracy of the sorting) from HD and urological cancer patients and cloned them. CD4^+^CD8αα and CD4^+^CD8αβ T cell clones were then stimulated for 48 h with anti-CD3/CD28 antibodies and supernatants were harvested to measure levels of antitumor Th1-type cytokines (IFN-γ, TNF-α), as well as of pro-tumor Th2-type cytokines (IL-4, IL-5). We expected that the latter cytokines may be highly produced by DP T cells as a high frequency of them expressed the Th2 surrogate marker CRTH2 (Chemoattractant Receptor-homologous Molecule Expressed on T Helper Type 2), compared to the low levels observed in CD4 T cells (Supplementary Figure 2). Indeed, DP T cell clones from patients were found to secrete levels of IFN-γ and TNF-α comparable to DP T cells from healthy individuals (Figure 4A). However, CD8αβ DP T cells from patients were found to secrete significantly higher amounts of both IL-4 and IL-5, as compared to HD (Figure 4B). CD8αα DP T cells from patients also secreted elevated levels of IL-5 (Figure 4B).

### Functional Role of DP T Cells Toward Polarization of Naïve CD4 T Cells

We first interrogated a putative immunosuppressive function of DP T cells toward CD4 and/or CD8 T-cell proliferation (17), but found no effect in a CFSE-based suppression assay (*data not shown*). Next, as DP T cells secrete cytokines that may favor Th2 development, we investigated the ability of supernatants from stimulated DP T cells to polarize naïve CD4 T cells into Th1 vs. Th2 functional profiles (Figure 4C). To this purpose, we used the above-mentioned supernatants of stimulated and non-stimulated DP T cells derived from healthy donors and patients with urological cancers. As depicted in Figure 4C, we exposed naïve CD4 T cells (CD45RA^+^CCR7^+^, sorted from healthy donors' blood) to those supernatants for 48 h, along with T-cell receptor stimulation (using anti-CD3/CD28 antibodies). Cells were then washed, transferred into new wells and incubated for 4 days in the presence of renewed DP T-cell supernatants. T-helper polarization of CD4 T cells was analyzed by intracellular cytokine staining (INF-γ and IL-4) following a 6 h unspecific re-stimulation (Figure 4D). Supernatants from stimulated DP T cells strongly increased the frequency of CD4 T cells expressing IL-4, while reducing the frequency of INF-γ-expressing cells (Figure 4E). As a result, the ratio between INF-γ- and IL-4-producing cells was dramatically reduced by activated DP T-cell supernatants (Figure 4F). CD8αα and CD8αβ DP T cells showed similar ability to shift this ratio (Supplementary Figure 3A). Interestingly, DP T cells from patients were even more prone to invert the INF-γ^+^/IL-4^+^ CD4 T-cell ratio (Figure 4G), which could be explained by a stronger ability to restrain the generation of CD4 T cell expressing INF-γ (Supplementary Figure 3B). These results suggest that DP T cells favor a Th2 over Th1 polarization of CD4 T helper cells. Accordingly, compared to HD, urological cancer patients showed a higher frequency of circulating Th2 CD4 T cells, as assessed by *ex vivo* CRTH2 expression (Supplementary Figure 3C).

## Discussion

Our results indicate that circulating CD4^+^CD8^+^ T cells are increased in patients with urologic cancers, and show a differentiation profile biased toward an effector memory phenotype. As expected, DP T cells were found to be high cytokine producers, but only type-2 cytokines were increased in patients DP T cells as compared to healthy donors. Notably, DP T cell were found to favor a Th2 polarization of CD4 T cells *in vitro*, at the expense of the protective Th1 functional profile known to play a crucial role in anti-tumor immunity. This newly identified ability of DP T cells was observed in healthy donors and exacerbated in patients with urological cancer.

DP T cells were already described in patients with particular lymphomas (19–21), melanoma (22), breast (23), colorectal cancer (24), and recently in renal cancer (25). In these contexts, DP T cells were often analyzed in tumor-infiltrating lymphocytes (e.g., after amplification and clonal expansion) but were more rarely quantified in the peripheral blood. Nevertheless, normal levels of circulating DP T cells were found in patients with melanoma and Hodgkin's lymphoma when compared to healthy donors (19, 22). In contrast, we found a consistent, yet moderate, increase in the frequency of circulating DP T cells in patients with all three urological cancers tested.

Mechanisms behind DP T-cell expansion can only be speculative as origin of circulating DP T cells—i.e., outside the thymus—remains unclear. Extrathymic DP T cells may originate from single-positive T cells under particular conditions, as CD4 expression could be acquired following activation of CD8 T cells (26) and conversely, CD4 T cells were proposed to co-express CD8 under continuous antigen stimulation in the presence of intestinal factors (27). However, analysis of the TCR Vβ usage by sclerosis- and melanoma-associated T cells suggested that DP T cells in these contexts may have a clonal origin distinct from that of conventional CD4 or CD8 single-positive T cells (22, 28). In the tumor from a patient with colorectal cancer, highest tumor-reactivity was observed within DP T cells from an overrepresented Vβ subset (24). Thus, elevated levels of DP T cells in cancer contexts may be related to clonal expansion of cognate DP T cells. This is in accordance with our observation that DP T cells from urological cancer patients showed elevated levels of effector memory cells, together with a reduced naive compartment. Of note, intra-tumor DP T cells were previously described as showing an effector memory phenotype (22, 23). How the tumor may directly or indirectly favor DP T-cell activation/expansion warrants further investigations. Of note, IL-9 was proposed as a survival and activation factor of DP T cells in melanoma tumors (29). However, a loss of IL-9-producing T cells was described in patients with metastatic melanoma (30). IL-9 was not studied in patients with urological cancers to date, yet absence of IL-9 mRNA was reported in bladder tumor cell lines (31). Besides, expansion of extrathymic DP T cells was reported in ≈20% of patients receiving radiotherapy for Hodgkin's disease (19). However, radiotherapy in patients with urological cancers (i.e., pelvic area) is unlikely to be involved in DP T-cell expansion, as it was suggested to reflect extrathymic T-cell development as a result of impaired thymic function following mediastinal irradiation. Altogether, the appearance of extrathymic DP T cells remains a puzzling question and future studies such as transcriptomic analyses are required to decipher their origin.

Beside reactivity toward viral antigens (EBV, Flu, HCV, HIV) (32, 33), DP T cells seem to show large tumor cross-reactivity (24), with one clone found to recognize various tumors. DP T cells were also proposed to recognize self-antigens, as suggested in melanoma or sclerosis patients (22, 28). In most reports in cancer contexts, DP T cells were consistently found to show MHC class I-restricted reactivity to autologous tumor cells, as assessed by cytokine production, markers of lytic capacity, or *bona fide* cytotoxic activity (20–24). As such, they could be considered as similar to single-positive CD8^+^ T cells, showing anti-tumor properties. However, the most notable attribute distinguishing DP T cells from conventional CD4 and CD8 single-positive T cells is their higher production of Th2 cytokines. Yet DP T cells also produce Th1 cytokines, but at similar levels compared to conventional T cells (22–24). Our data confirmed the general ability of DP T cells to produce Th2 cytokines and additionally showed that this production was enhanced in DP T cells derived from urological cancer patients. It could be speculated that tumors instruct dendritic cells to favor Th2-cytokine production by DP T cells, as already known for CD4 T cells (34, 35). Indeed, the tumor microenvironment support polarization of type-2 immune cells, not only in T cells but also in macrophages (i.e., M2) (36) and innate lymphoid cells (i.e., ILC2), as we showed in patients with bladder cancer (11).

Important heterogeneity exists in the function of DP CD4^+^CD8^+^ T cells, which seems to depend on the expression of the CD8α or β subunit (10). Albeit conflicting results have been reported about the cytotoxic capacity of CD4^+^CD8αβ DP T cells, they seem to have low cytotoxic anti-tumor function (10, 23, 24, 32), which can be increased in the presence of IL-9 (29). Besides, CD4^+^CD8αβ DP T cells may exert helper functions through the expression of CD40L (37). In contrast, CD4^+^CD8αα DP T cells may have an immunosuppressive role. Indeed, colonic bacteria-induced DP T cells, expressing CCR6 and CXCR6, were shown to produce IL-10 and inhibit T-cell proliferation via CD39 activity, similarly to Tregs (17, 38). However, we did not observe any direct suppressive function when using DP T cells clones, either CD8αα or CD8αβ, isolated from PBMC of healthy donors or cancer patients. Since CD8αα DP T cells with regulatory properties have been identified so far only in colonic mucosa from HD and patients with inflammatory bowel disease (17, 38), further investigations are needed to clarify whether a subset with direct suppressive function may also be found in cancer patients. Nevertheless, we asked whether and how DP T cells may influence CD4^+^ T-cell function. Our data demonstrated that soluble factors secreted by DP T cells are able to favor polarization of naïve CD4^+^ T cells into a Th2 profile, while restraining Th1 differentiation. Interestingly, this effect was observed with DP T cells from both healthy donors and patients, but was even more pronounced when using patients-derived DP T cells. This could be explained by the higher effector memory profile associated with higher Th2-type cytokine production observed in DP T cells from patients, when compared to healthy donors.

It would be of interest to target DP T cells in order to improve anti-tumor type-1 immunity. However, direct targeting of DP T cells may prove to be difficult due to their existence as a T-cell development stage and to the lack of a specific marker able to distinguish them from the indispensable single-positive T cells. Moreover, removal of DP T cells may not be relevant since, as discussed above, they also show a beneficial cytotoxic effect toward tumor cells. It may thus be of interest to specifically target their pro-tumor activities, i.e., the secretion of Th2 cytokines of which DP T cells seem to be a major producer as highlighted in this and other studies (22–24). To this regard, IL-4 blockade in mouse tumor models was shown to reduce immunosuppressive cells, enhance anti-tumor cytotoxic T-cell activity and thus delay tumor progression, as well as synergistically improve immunotherapies (39).

Altogether, DP T cells from patients with urological cancers are not only increased but particularly prone at favoring a Th2 profile at the expense of Th1 cells, suggesting a deleterious role with regard to effective antitumor immunity in these patients. DP T cells and conventional Th2 cells may fuel each other, promoting a subverted immune contexture allowing urological tumor escape.

## Data Availability

All datasets generated for this study are included in the manuscript and/or the Supplementary Files.

## Author Contributions

LD: conception and design; PB, MC, LD, VC, IL, RB, TT, MV, S-CR-D, FD, and PJ: acquisition of data; PB, MC, LD, and DN-H: analysis and interpretation of data; PB, MC, LD, DN-H, IL, RB, TT, MV, and PJ: writing, review and/or revision of the manuscript; VC, S-CR-D, FD, and LD: administrative, technical, or material support; LD and PJ: study supervision.

### Conflict of Interest Statement

The authors declare that the research was conducted in the absence of any commercial or financial relationships that could be construed as a potential conflict of interest.

## Supplementary Material

Supplementary Figure 1 Similar frequency of single-positive and total T cells in HD and urological cancer patients. **(A)** Representative gating strategy for the identification of DP T cells from PBMC of prostate cancer patient. **(B)** Frequency of conventional CD4^+^ or CD8^+^ single positive cells among T cells from HD and urological cancer patients. **(C)** Frequency of total CD3^+^ T cells among live PBMC. Data are expressed as mean ± SEM.

Supplementary Figure 2 Overexpression of CRTH2 on the DP T cells. CRTH2 expression (frequency, mean ± SEM) by indicated T-cell populations in PBMC from HD and urological cancer patients.

Supplementary Figure 3 T helper polarization by DP T cell subsets. **(A)** Ratio between IL-4 and IFN-γ expression by polarized CD4^+^ naïve T cells (from 2 HD) upon stimulation in the presence of supernatants from CD8αα and CD8αβ DP T cell clones from HD or patients. **(B)** Variation in the percentage of indicated cytokine-expressing CD4^+^ T cells upon conditioning with supernatants from stimulated DP T-cell clones from HD and patients. **(C)** CRTH2 expression (frequency, mean ± SEM) by CD4 T cells in PBMC from HD and urological cancer patients. ^*^*p* ≤ 0.05; ^**^*p* ≤ 0.01; ^****^*p* ≤ 0.0001.
