# Supplementary figures and images for: Double Positive CD4+CD8+ T Cells Are Enriched in Urological Cancers and Favor T Helper-2 Polarization
Source: Front Immunol. 2019 Mar 29;10:622. doi: 10.3389/fimmu.2019.00622 (PMC6450069; doi:10.3389/fimmu.2019.00622)

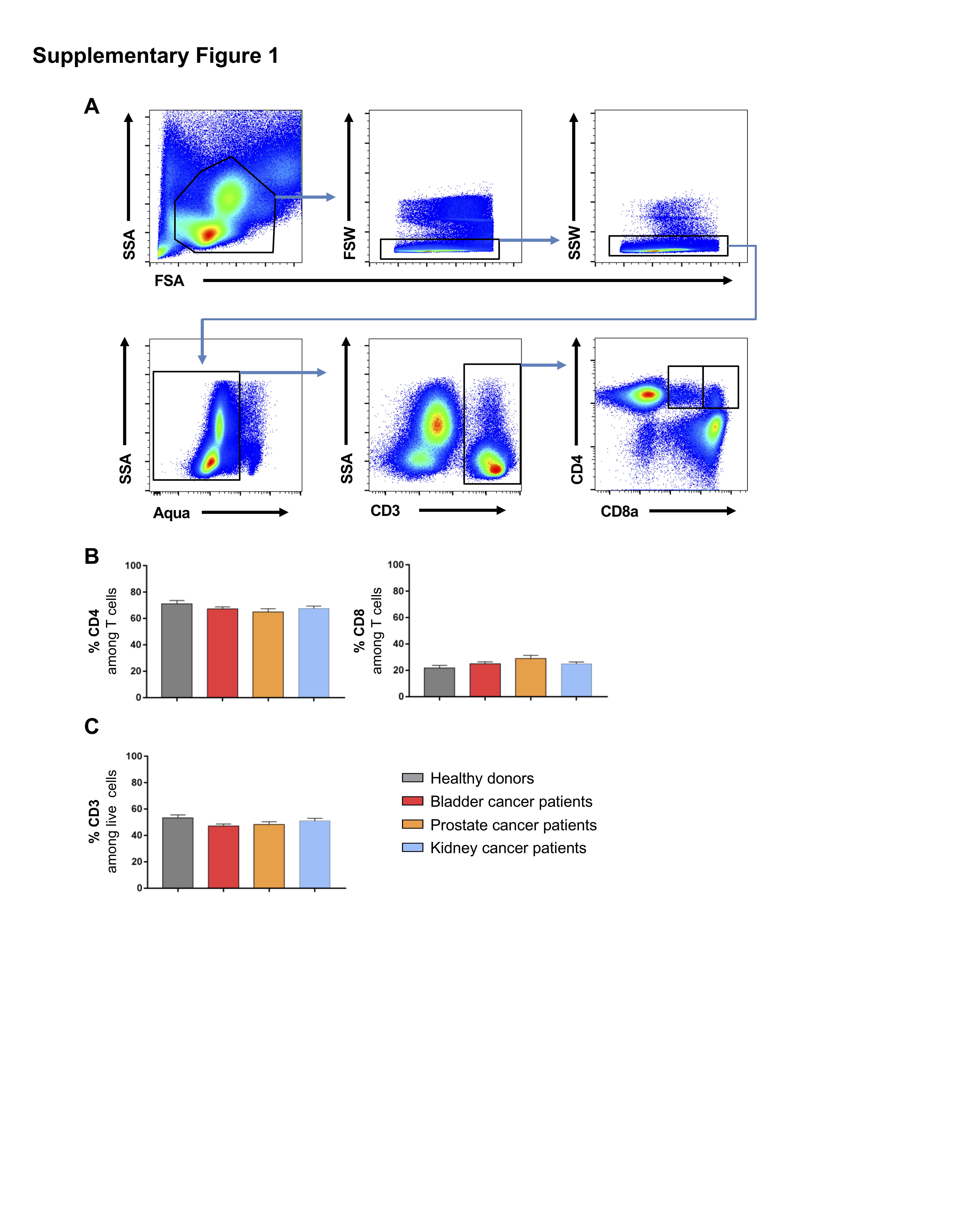

Supplement: Supplementary Figure 1 — Similar frequency of single-positive and total T cells in HD and urological cancer patients. (A) Representative gating strategy for the identification of DP T cells from PBMC of prostate cancer patient. (B) Frequency of conventional CD4+ or CD8+ single positive cells among T cells from HD and urological cancer patients. (C) Frequency of total CD3+ T cells among live PBMC. Data are expressed as mean ± SEM. [file Image_1.JPEG]

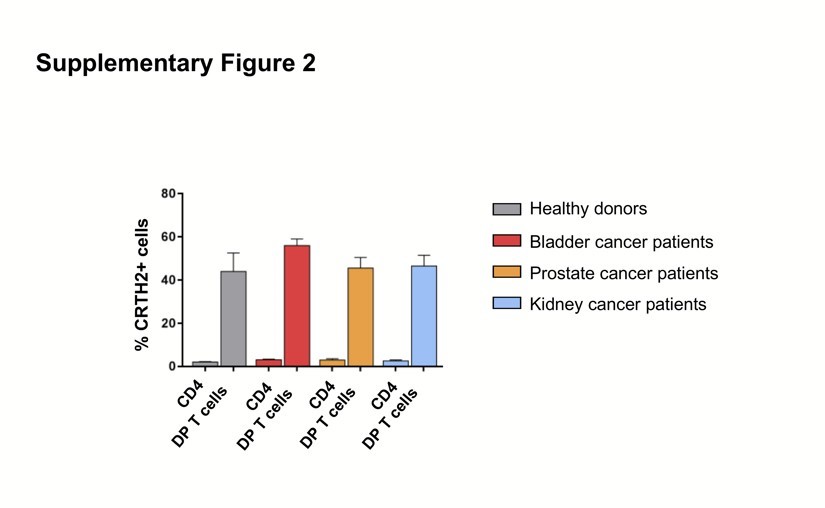

Supplement: Supplementary Figure 2 — Overexpression of CRTH2 on the DP T cells. CRTH2 expression (frequency, mean ± SEM) by indicated T-cell populations in PBMC from HD and urological cancer patients. [file Image_2.jpg]

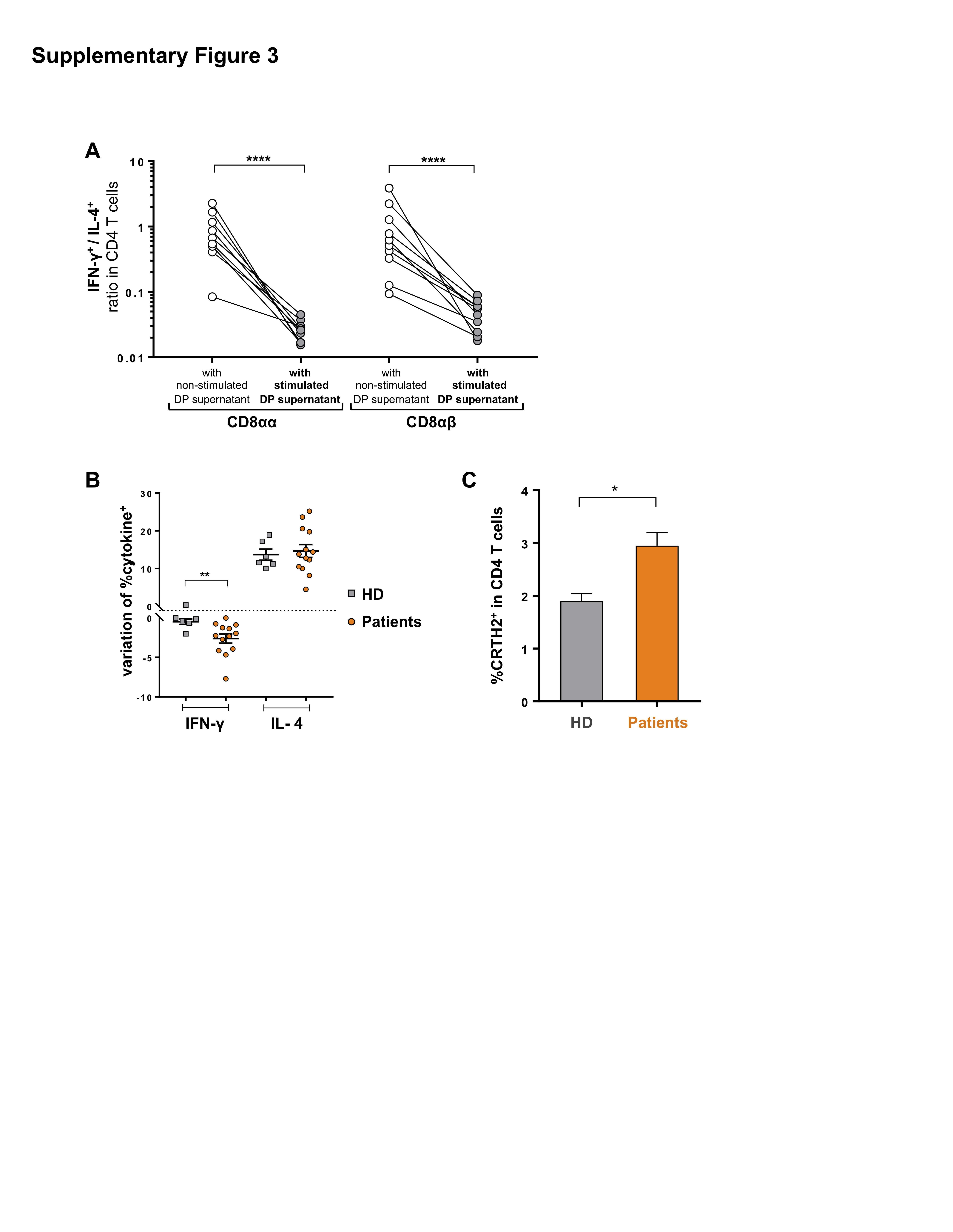

Supplement: Supplementary Figure 3 — T helper polarization by DP T cell subsets. (A) Ratio between IL-4 and IFN-γ expression by polarized CD4+ naïve T cells (from 2 HD) upon stimulation in the presence of supernatants from CD8αα and CD8αβ DP T cell clones from HD or patients. (B) Variation in the percentage of indicated cytokine-expressing CD4+ T cells upon conditioning with supernatants from stimulated DP T-cell clones from HD and patients. (C) CRTH2 expression (frequency, mean ± SEM) by CD4 T cells in PBMC from HD and urological cancer patients. *p ≤ 0.05; **p ≤ 0.01; ****p ≤ 0.0001. [file Image_3.JPEG]
